# Prognostic value of tumor-infiltrating CD163^+^macrophage in patients with metastatic gastric cancer undergoing multidisciplinary treatment

**DOI:** 10.1186/s12885-022-09713-y

**Published:** 2022-06-03

**Authors:** Jun Kinoshita, Sachio Fushida, Takahisa Yamaguchi, Hideki Moriyama, Hiroto Saito, Mari Shimada, Shiro Terai, Koichi Okamoto, Keishi Nakamura, Itasu Ninomiya, Shintaro Yagi, Noriyuki Inaki

**Affiliations:** 1 Department of Gastrointestinal Surgery, Graduate School of Medical Science, Kanazawa University, 13-1 Takara-machi, Kanazawa, Ishikawa, 920-8641 Japan; 2 Department of Hepato-Biliary-Pancreatic Surgery, Graduate School of Medical Science, Kanazawa University, 13-1 Takara-machi, Kanazawa, Ishikawa, 920-8641 Japan

**Keywords:** Metastatic gastric cancer, CD163^+^macrophage, Conversion surgery

## Abstract

**Background:**

The multidisciplinary treatment including induction chemotherapy plus conversion surgery (CS) has attracted attention as a new strategy to improve the outcome of metastatic gastric cancer (MGC). However, it is unclear which patients achieve a good response to chemotherapy and successful CS. Tumor-infiltrating immune cells (TIICs) have been reported to be both prognostic and predictive biomarkers not only in immunotherapy but also in chemotherapy in many cancer types. However, there have been no reports on the usefulness of TIICs as biomarkers in conversion surgery for MGC. The aim of the present study was to evaluate the association between the TIICs and treatment outcome for the multidisciplinary treatment in MGC.

**Methods:**

We retrospectively analyzed 68 MGC patients who received docetaxel plus cisplatin plus S-1 (DCS) therapy between April 2006 and March 2019 in our institute. The number of tumor-infiltrating CD4^+^, CD8^+^, Foxp3^+^lymphocytes, CD68^+^, CD163^+^macrophages in pre-treatment endoscopic biopsy samples were evaluated to investigate their predictive value for multidisciplinary treatment.

**Results:**

Fifty patients underwent CS following DCS therapy (CS group), whereas 18 patients underwent DCS therapy alone (non-CS group). The median survival time (MST) of CS group was 33.3 months, which was significantly longer than the MST of 9.0 months in non-CS group (*p* < 0.01). The number of CD163^+^macrophages was extracted as an independent prognostic factor for overall survival in all patients. There were more cases of high infiltration of CD163^+^macrophages in non-CS group than in CS group. Furthermore, in CS group, pathological responders to DCS therapy showed low infiltration of CD163^+^ macrophages, and high infiltration of CD8^+^lymphocyte. CD163 low group showed a significant prolonged survival compared with CD163 high group in patients who underwent CS (*p* = 0.02).

**Conclusions:**

The pre-treatment CD163^+^macrophages infiltration would be a pivotal biomarker for predicting prognosis and pathological response to multidisciplinary treatment among TIICs in MGC.

Thus, for patients with low CD163^+^macrophage infiltration in pre-treatment biopsy sample, diagnostic imaging should be performed frequently during chemotherapy to avoid missing the optimal timing for CS, and CS should be aggressively considered as a treatment option if curative resection is deemed feasible.

## Background

The prognosis for patients with metastatic gastric cancer (MGC) remains insufficient, and palliative chemotherapy with best supportive care is the mainstay for this cohort. However, the development of highly effective regimens using new anticancer drugs has led to remarkable tumor shrinkage and, even in MGC, some patients with good response to chemotherapy have been able to undergo curative resection and survive for long periods. This strategy is referred to as “conversion surgery” (CS), which is defined as surgical treatment aimed at R0 resection after successful chemotherapy for tumors that were initially unresectable or marginally resectable [1]. We previously reported the efficacy of CS following docetaxel plus cisplatin plus S-1 (DCS) therapy in MGC patients [2]. Furthermore, large amounts of historical data have demonstrated the benefits of CS for MGC in select patient groups [3]. Thus, this multidisciplinary treatment including induction chemotherapy plus CS is a promising new treatment option for MGC patients with non-curable factors, who were considered to have little hope of a cure.

Importantly, in MGC, there are a wide variety of biological characteristics thought to differ depending on each metastatic condition. Recently, Yoshida et al. created a comprehensive classification of CS with respect to the biological and heterogeneous characteristics of stage IV GC [4]. In this classification, patients without macroscopic peritoneal metastasis were divided into two categories as category 1 and 2. Category 1 included patients with a single liver metastasis, patients with positive cytology, or patients with a limited para-aortic lymph node metastasis. For patients in category 2, metastases were considered technically or oncologically unresectable, and this category represented a more extensive metastatic situation than category 1. In contrast, categories 3 and 4 constituted patients with macroscopic peritoneal metastasis, who have low sensitivity to conventional chemotherapy and are the most refractory population in stage IV GC [5]. Accordingly, operations are defined as cytoreductive surgery rather than CS for patients with peritoneal metastasis [4]. Thus, patients in category 1, 2 are considered to be the best candidates for CS in the current therapeutic management. However, CS is not an option for all category 1 and 2 patients, and it is necessary to consider how to select a population to maximize the efficacy of surgical intervention. CS should be considered for MGC patients who have responded well to chemotherapy and for whom surgical intervention offers curative potential [1, 6]. However, because it is unclear which patients respond to chemotherapy and succeed in CS, the development of surrogate markers that reflect heterogeneity in both clinical and oncologic features of MGC patients is critical in planning treatment strategies for this cohort.

Tumor-infiltrating immune cells (TIICs) are indispensable components of the tumor microenvironment and have recently gained consideration as both prognostic and predictive biomarkers of novel therapeutic targets in some solid tumors [7, 8]. Tumor infiltrating lymphocytes (TILs) are the most extensively studied immune cell population, particularly in breast cancer, as a robust prognostic tool [9]. In addition to TILs, macrophages within tumors (tumor-associated macrophages [TAMs]) are another immune cell population that has been shown to have key homeostatic functions that influence tumor maintenance and growth. Macrophages show functional plasticity in response to the immune environment and have mainly two distinct phenotypes: the pro-inflammatory M1 phenotype and the anti-inflammatory M2 phenotype. Most TAMs within the tumor microenvironment exhibit the M2-like phenotype and promote tumor growth and metastasis via different mechanisms [10, 11]. CD68 has been commonly used as a macrophage marker for both M 1 and M 2 macrophages. CD163, a hemoglobin/haptoglobin complex scavenger receptor expressed almost exclusively on circulating monocytes and tissue macrophages, has been recognized as a valuable specific marker of M2 TAMs [12, 13]. Increased presence and density of TAMs has been reported to correlate with poor prognosis and resistance to treatment [14]. However, whether quantifying immune cells in the tumor microenvironment effectively identifies patient responses to multidisciplinary treatment for MGC remains to be elucidated. In the present study, we investigated the potential value of TIICs in pre-treatment biopsy specimens as a biomarker for predicting response to chemotherapy and survival in MGC, compared with conventional clinicopathological prognostic factors for CS.

## Methods

### Patients

We retrospectively identified patients who were diagnosed with metastatic gastric adenocarcinoma and received DCS therapy as induction chemotherapy at our institution between April 2006 and March 2019. The inclusion criteria for this study were as follows: (1) first-time case of gastric adenocarcinoma pathologically diagnosed by upper gastrointestinal endoscopic biopsy; (2) clinically diagnosed as stage IV with distant metastatic lesions according to the Japanese Classification of Gastric Carcinoma, 3rd English edition[15]; (3) diagnosed without peritoneal dissemination by CT imaging or staging laparoscopy; (4) patients who received at least one cycle of DCS therapy as induction chemotherapy; (5) sufficient pre- chemotherapy tumor samples for immunohistochemical staining; (6) no prior chemotherapy, radiotherapy, or major surgical procedures; and (7) patients without concurrent multiple cancers. A total of 68 patients were included in the current study. Medical data such as clinicopathological features, treatment effects, surgical findings, and clinical course were extracted retrospectively from our hospital database. Written informed consent was obtained from all patients as required by the Kanazawa University Review Board (Kanazawa, Japan) for the use of their clinical and pathological data.

All laboratory data were obtained from routine blood test at the time of diagnosis. NLR (neutrophil-to-lymphocyte ratio) was calculated by dividing the number of neutrophils by the number of lymphocytes; LMR (lymphocyte-to-monocyte ratio) was calculated by dividing the number of lymphocytes by the number of monocytes; and PLR (platelet-to-lymphocyte ratio) was calculated by dividing the number of platelets by the number of lymphocytes.

### DCS regimen for induction chemotherapy

DCS therapy consists of 35 mg/m2 of docetaxel and 35 mg/m2 of cisplatin administered intravenously on days 1 and 15, and 80 mg/m2 of S-1 administered orally twice daily for 14 days, followed by 14 days of rest within one cycle [16]. The clinical response of measurable metastatic tumors was assessed according to the guidelines of the Response Evaluation Criteria in Solid Tumors (RECIST) guidelines, version 1.1. [17]. When distant metastases were controlled or disappeared during chemotherapy, the indication for CS was determined according to the status of residual disease. In this study, CS was defined as surgery with curative intent for R0 resection [2].

### Immunohistochemistry for TIICs

Full sections of pretreatment biopsy specimens from the primary tumor were used to evaluate TIIC numbers. Only tissues with sufficient tumor components were selected for further study. Briefly, formalin-fixed, paraffin-embedded (FFPE) tissue blocks were cut into 4-μm sections, deparaffinized in xylene, and rehydrated in a graded series of ethanol. Blocking was performed with protein block serum (DakoCytomation, Kyoto, Japan) for 10 min at room temperature, followed by the addition of normal goat serum. Next, the slides were incubated with primary rabbit polyclonal anti-CD4 antibody (1:300 dilution; Abcam, Cambridge, UK), primary rabbit polyclonal anti-CD8 antibody (1:200 dilution; Abcam), primary mouse monoclonal anti-Foxp3 antibody (1:100 dilution; Abcam), primary rabbit polyclonal anti-CD163 antibody (1:300 dilution; Bioss, Woburn, USA), and primary mouse monoclonal anti-CD68 antibody (1:200 dilution; Abcam) at 4℃ overnight. The tissues were then immersed in an EnvisionTM polymer solution (horseradish peroxidase; Dako) for 1 h. Specific antigen–antibody reactions were comfirmed using 0.2% diaminobenzidine tetrahydrochloride and hydrogen peroxide. Sections were examined using a microscope (Olympus, Tokyo, Japan). For each tissue specimen from the cancer lesions, five randomized fields of positively-stained cells were counted under a high-power field (HPF) of × 200 magnification. Positive cell counts were calculated and confirmed by two investigators blinded to clinicopathological features.

### The histological response to chemotherapy

The pathological response was evaluated by a pathologist at Kanazawa University Hospital using the surgically-excised specimens. The histopathological response was categorized according to the criteria of the Japanese Classification of Gastric Carcinoma, 3rd English edition [15]. The following five categories were defined by the viable residual tumor cells in the entire cancer tissue: Grade 3, no viable residual tumor cells: Grade 2, few residual tumor cells; Grade 1b, less than 2/3 residual tumor cells; Grade 1a, more than 2/3 residual tumor cells: and Grade 0, no response to chemotherapy. In the current study, patients with grade 0, 1a, and 1b were defined as non-responders, and patients with grade 2 and 3 were defined as responders [15].

### Statistical analysis

The association between immunohistochemical variables and clinicopathological features were investagated by Chi-square or Fisher's exact test. The Kaplan–Meier method was used to estimate the survival curve, and the log-rank test was used for survival analysis. Optimal cut-off values for each TIIC, NLR, LMR, PLR, and albumin were calculated using receiver operating characteristic (ROC) curve analysis with maximal Youden index value [18]. ROC curve was analyzed with OS data of total patient population and determine their prognostic predictive value. Only significant variables in univariate Cox regression analysis (*P* < 0.05) were subjected to multivariate Cox regression analysis with stepwise selection to identify the independent prognostic factors. Statistical analyses were performed using SPSS 19.0 (IBM SPSS Inc., Armonk, NY) and R version 3.1.2 (R Foundation for Statistical Computing, Vienna, Austria). Statistical significance was set at a two-sided *p* < 0.05.

## Results

### Overall survival of metastatic GC patients who underwent DCS therapy

​ The median survival time (MST) of all 68 patients enrolled in this study was 24.8 months, with 3-year overall survival (OS) of 40.0% and 5-year OS of 35.6%. Of these 68 patients, 50 patients underwent CS with curative intent following DCS therapy (CS group), whereas 18 patients underwent DCS therapy alone (non-CS group). The MST of the CS group was 33.3 moths, which was significantly longer than the MST of 9.0 months in the non-CS group (*p* < 0.0001).

### Patient characteristics in the non-CS group compared to the CS group

**Fig. 1:**
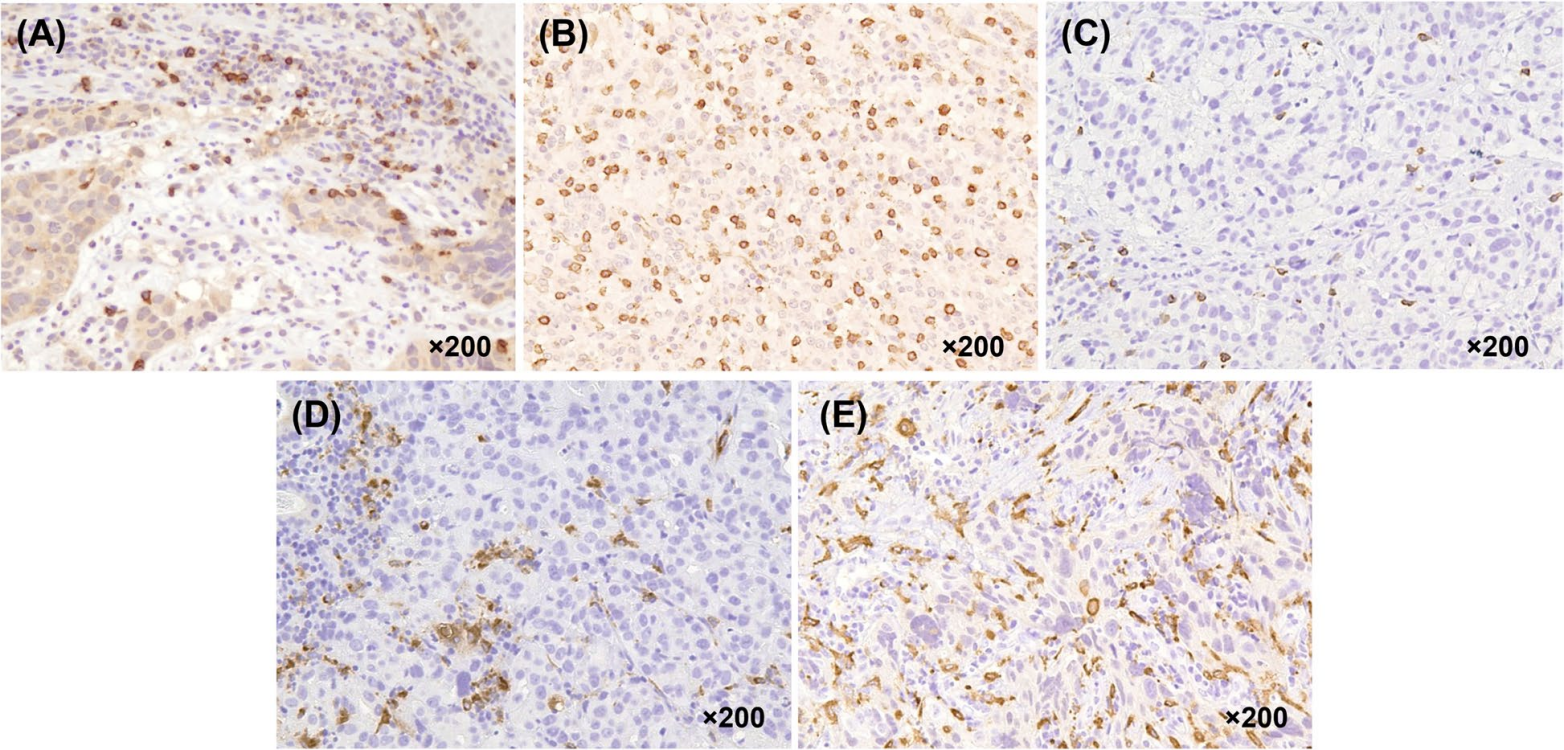
Representative immunohistochemical images for tumor infiltrating imuune cells in primary tumor of biopsy samples. **a** CD4, **b** CD8, **c** Foxp3, **d** CD68, **e** CD163. Magnification, × 200

**Fig. 2:**
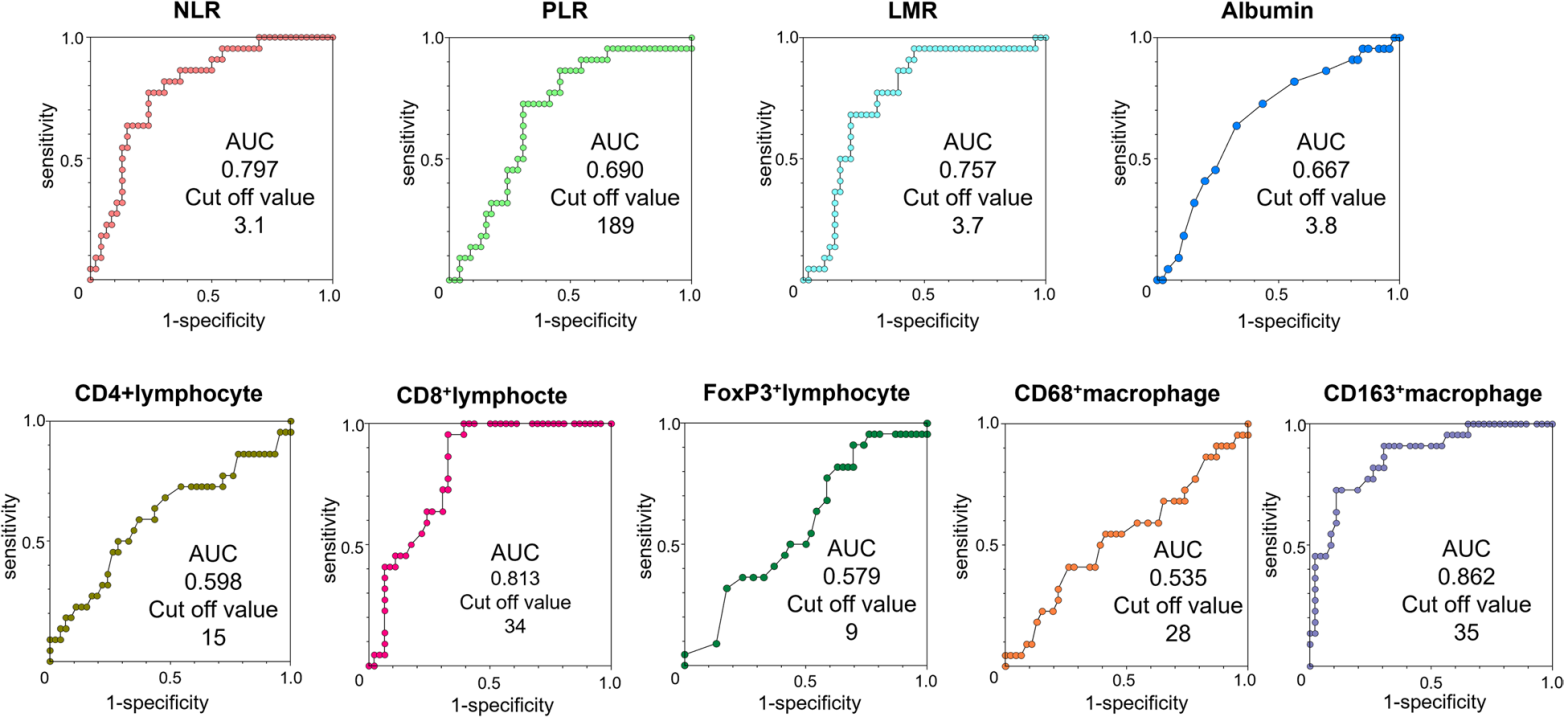
The receiver operating curves of individual parameters. The optimal cut-off levels and areas under the curves of NLR, LMR, PLR, albumin, CD4^+^lymphocyte, CD8^+^lymhpocte, FoxP3^+^lymphocyte, CD68^+^macrophage, and CD163^+^macrophage were calculated by ROC curve

We investigated the differences in clinicopathological characteristics before chemotherapy and the TIICs in the primary tumor pre-treatment biopsies between the CS group and the non-CS group. Figure 1 is a representative image of immunohistochemical staining of TIICs. Figure 2 showed the optimal cut-off levels and areas under the curves (AUCs) of NLR, LMR, PLR, albumin, and TIICs calculated by ROC curve. As shown in Table 1, with respect to the clinicopathological factors, the non-CS group had significantly more patients with multiple non-healing factors, Yoshida classification 2, higher NLR, and lower LMR than the CS group. Regarding the TIICs, there were more cases of high infiltration of CD163^+^macrophages in the non-CS group than in the CS group. However, the numbers of CD4^+^lymphocytes, CD8^+^lymphocytes, Foxp3^+^lymphocytes, and CD68^+^macrophages were not significantly different between the two groups.

**Table 1:** Comparison of clinicopathological characteristics between non-conversion surgery group and conversion surgery group

	**cut off level**	**non-conversion surgery group (*n* = 18)**	**conversion surgery group (*n* = 50)**	***p***-value
Sex (M/F)		15/8	41/9	0.115
Age (years)		63.5(30–78)	66.5(25–75)	0.928
Performance Status 0/1		13/5	44/6	0.119
Tumor location (U/M/L/whole)		8/7/3/0	19/18/13/0	0.719
Macroscopic type (0/1/2/3/4/5)		0/1/6/11/0/0	0/2/24/24/0	0.560
Histological differentiation intestinal/diffuse		5/13	22/28	0.228
Distant metastatic site				
Hematogenous +/-		7/11	18/32	0.827
Lymphogenous +/-		12/6	36/14	0.670
Multiple non-curative factors +/-		6/12	33/17	0.016
Yoshida’s Category 1/2		0/18	23/27	< 0.001
Albumin (g/dl) low/high	3.8	12/6	25/25	0.223
CEA (ng/ml) low/high	5	8/10	22/28	0.974
CA19-9 (U/ml) low/high	37	5/13	20/30	0.356
NLR low/high	3.1	3/15	28/22	0.004
PLR low/high	189	7/11	26/24	0.340
LMR low/high	3.7	13/5	20/30	0.019
CD4^+^ lymphocyte /Hpf low/high	15	9/9	21/29	0.557
CD8^+^ lymphocyte /Hpf low/high	34	16/2	38/12	0.246
Foxp3^+^lymphocyte /Hpf low/high	9	8/10	22/28	0.974
CD68^+^ macrophage /Hpf low/high	28	7/11	25/25	0.418
CD163^+^ macrophage /Hpf low/high	35	3/15	25/25	0.013

*NLR* Neutrophil to lymphocyte ratio, *PLR* Platelet to lymphocyte ratio, *LMR* Lymphocyte to monocyte ratio, *Hpf* High power field

### Association between pre-treatment clinicopathological parameters and OS

In order to evaluate the prognostic potential of clinicopathological factors assessable prior to chemotherapy in all patients enrolled in this study, univariate and multivariate analyses using a COX proportional hazard model were performed. In univariate analysis, no significant differences were observed in the performance status, multiple non-curative factors, or the type of metastasis, but significant differences were found in Yoshida classification, serum albumin, NLR, LMR, CD8^+^lymphocyte infiltration, and CD163^+^macrophage infiltration (Table 2). Next, we performed multivariate analysis to determine the independent factors impacting OS. Variables with *p*-values < 0.05 in the univariate analysis—Yoshida classification, NLR, LMR, serum albumin, CD8^+^lymphocytes, and CD163^+^macrophages—were included in the multivariate analysis. To avoid collinearity between NLR and LMR, two similar multivariate analyses were performed separately, as shown in Table 3. The results showed that the number of infiltrating CD163^+^macrophages was an independent prognostic factor for OS in both analyses (hazard ratio [HR] = 2.281, 95% confidence interval [CI] = 1.017– 5.117, *p* = 0.045; HR = 1.945, 95% CI = 1.018–3.713, *p* = 0.043, respectively).

**Table 2:** The correlation between OS and clinicopathological factors assessable prior to chemotherapy in all patients

Variables (*n* = 68)	Cut off level	Number of patients	HR	95%CI	*p*
Sex male/female		56/12	0.671	0.318- 1.413	0.293
Age < 70 years/ ≥ 70 years/		52/16	1.211	0.592- 2.474	0.600
Performance status 0/1		57/11	1.503	0.665- 3.394	0.327
Histological differentiation intestinal /diffuse		41/27	1.859	0.927- 3.727	0.081
Haematogenous +/-		29/39	1.742	0.938- 3.230	0.078
Lymphogenous +/-		47/21	0.568	0.302- 1.068	0.079
Yoshida classification category 1/2		23/45	2.595	1.261–5.341	0.011
Multiple non-curative factors +/-		39/29	1.707	0.921–3.164	0.089
Albumin (g/dl) low/high	3.8	37/31	0.447	0.233- 0.853	0.015
CEA (ng/ml) low/high	5	38/30	0.719	0.384- 1.343	0.301
CA19-9 (U/ml) low/high	37	43/25	1.392	0.745- 2.598	0.299
NLR low/high	3.1	31/37	2.142	1.134- 4.047	0.018
PLR low/high	189	33/35	0.092	0.914- 3.207	0.092
LMR low/high	3.7	32/36	0.479	0.257- 0.892	0.020
CD4^+^ lymphocyte /Hpf low/high	15	29/39	0.610	0.329–1.129	0.116
CD8^+^ lymphocyte /Hpf low/high	34	54/14	0.324	0.126–0.832	0.019
Foxp3^+^ lymphocyte /Hpf low/high	9	47/21	1.671	0.886- 3.150	0.112
CD68^+^ macrophage /Hpf low/high	28	36/32	1.593	0.856- 2.964	0.142
CD163^+^ macrophage /Hpf low/high	35	28/40	3.459	1.725–6.937	0.001

*NLR* Neutrophil to lymphocyte ratio, *PLR* Platelet to lymphocyte ratio, *LMR* Lymphocyte to monocyte ratio

**Table 3:** Multivariate analysis of independent risk factor for overall survival in patients who underwent DCS therapy

	HR	95%CI	***p***
Multivariate analysis with NLR			
Yoshida classification category 1/2	1.652	0.774–3.524	0.193
Albumin	0.522	0.369–1.656	0.522
NLR	1.720	0.897–3.295	0.102
CD8^+^ lymphocyte	0.589	0.211–1.640	0.311
CD163^+^ macrophage	2.281	1.017–5.117	0.045
Multivariate analysis with LMR			
Yoshida classification category 1/2	1.980	0.915- 4.278	0.082
Albumin	0.842	0.397- 1.784	0.654
LMR	0.482	0.212–1.095	0.081
CD8^+^ lymphocyte	0.555	0.197- 1.563	0.265
CD163^+^ macrophage	1.945	1.018–3.713	0.043

*95%CI* 95% confidence interval, *HR* Hazard ratio, *NLR*, Neutrophil to lymphocyte ratio, *LMR* Lymphocyte to monocyte ratio

### The correlation between CD163^+^ macrophage infiltration and clinicopathological features

We investigated the correlation between CD163^+^macrophage infiltration and clinicopathological factors in all cases, as shown in Table 4. Patients with high infiltration of CD163^+^macrophages (CD163-high group) had significantly higher NLR, lower LMR, and lower albumin than those with low infiltration of CD163^+^macrophages (CD163-low group). In the CD163-high group, there was a tendency toward a higher proportion of category 2 patients, but the difference was not significant (*p* = 0.067).

**Table 4:** Associations between CD163^+^ macrophages infiltration and clinicopathological variables in all cases

Variables	CD163 low (*n* = 28)	CD163 high (*n* = 40)	*p*-value
Age, years	66.0(47–78)	67.0(30–78)	0.992
Sex, n(%)	23/5	33/7	0.970
Performance status	25/3	32/8	0.306
Histological differentiation Intestinal/diffuse	17/11	22/18	0.639
Multiple non-curative factors -/+	19/9	20/20	0.143
Yoshida’s category 1/2	13/15	10/30	0.067
Hematogenous -/+	17/11	20/20	0.382
Lymphogenous -/+	6/22	15/25	0.158
Albumin < 3.8 / ≥ 3.8	7/21	30/10	0.001 <
NLR < 3.1 / ≥ 3.1	17/11	15/25	0.008
PLR < 188.7 / ≥ 188.7	16/12	17/23	0.234
LMR ≥ 3.7 / < 3.7	9/19	23/17	0.039

*NLR* Neutrophil to lymphocyte ratio, *PLR* Platelet to lymphocyte ratio, *LMR* Lymphocyte to monocyte ratio

### Postoperative prognostic factors in patients who underwent CS

In the next phase, we examined the correlation between pT grade (pT3/pT4), pN grade (pN ≤ 1/pN ≥ 2) R (R0/R1, R2), and pathological response (grade ≤ 1b/grade ≥ 2), which are surgical or pathological prognostic factors that could be assessed after surgery, and survival in 50 patients undergoing CS. In the univariate analysis, a significant difference was observed in OS for the pN grade, R0, and pathological response (*p* = 0.025, 0.049, and 0.004, respectively), which are generally considered prognostic predictors of CS. Next, we performed a multivariate analysis for the variables with *p* < 0.05 in the univariate analysis to determine the independent factors impacting OS. The results showed that pathological response grade > 2 was an independent prognostic factor for OS (HR = 0.341, 95% CI = 0.134–0.868, *p* = 0.024).

### Association between pathological response and the number of TIICs

**Fig. 3:**
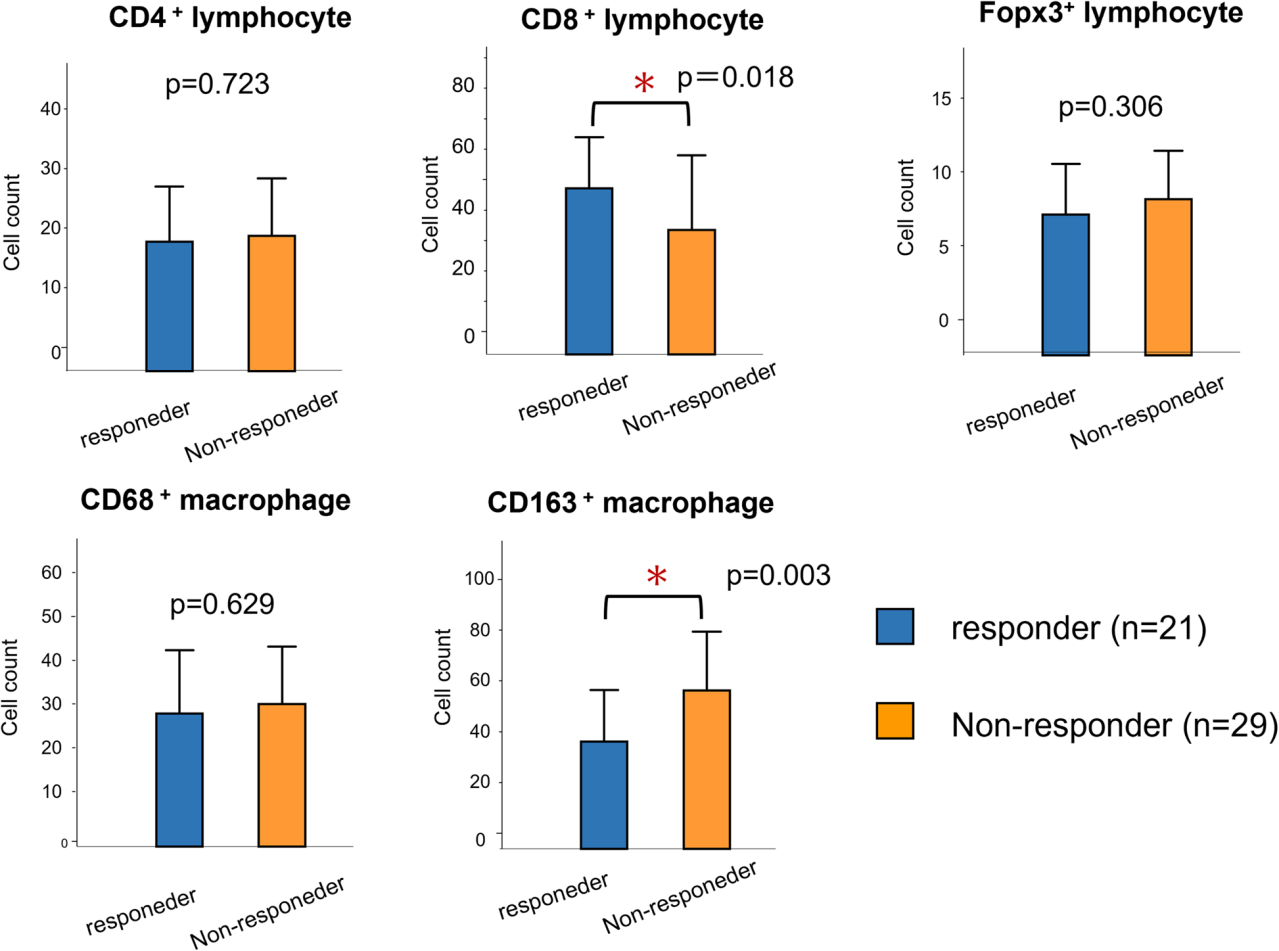
The association between the pathological response and the number of tumor infiltrating immune cells. Non-responder cases ranged from grade 0 to 1b, with a total of 29 patients. Responder cases were grade 2 or 3, with a total of 21 patients

We compared the number of TIICs between responder and non-responder groups in 50 patients who underwent CS. As shown in Fig. 3, the number of CD8^+^lymphocyte infiltrates was significantly higher, and the number of CD163^+^macrophage infiltrates was significantly lower, in the responder group. In contrast, the numbers of CD4^+^lymphocytes, Foxp3^+^lymphocytes, and CD68^+^macrophages were not significantly different between the two groups.

### OS according to the infiltration of CD163^+^ macrophages

**Fig. 4:**
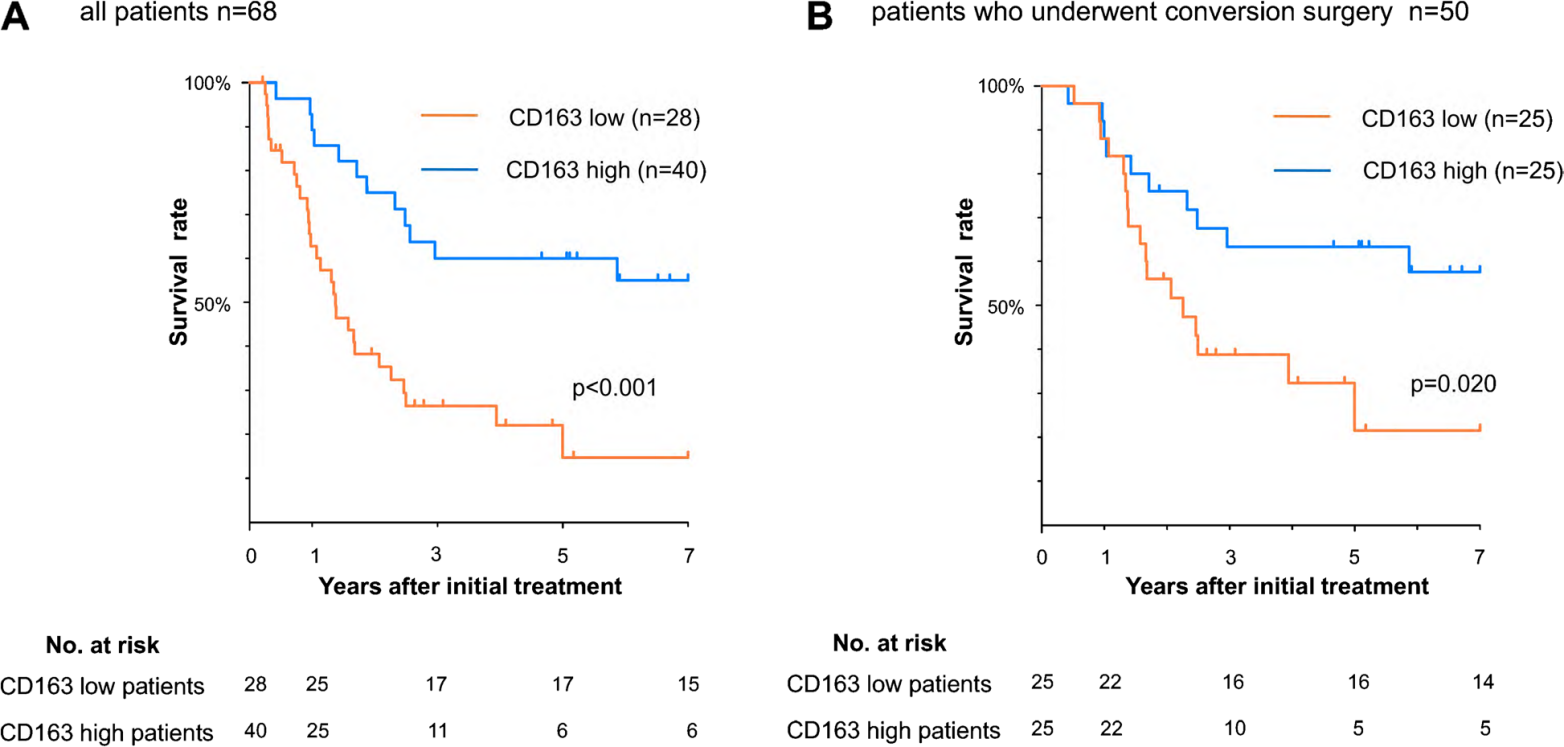
Kaplan–Meier curves of overall survival according to the infiltration of CD163^+^M2 macrophages. **a** in all patients enrolled in this study **b** in patients who underwent conversion surgery. Patients with high CD163^+^M2 macrophages infiltration had a significantly worse prognosis than those with low CD163^+^M2 macrophage infiltration in both all patients and conversion cases

Finally, we examined OS in the CD163-high group and CD163-low group with Kaplan–Meier curves. For all 68 patients enrolled in the study, OS in the CD163-low group, whose MST was not reached and whose 5-yr survival rate was 59.5%, was significantly longer than that in the CD163-high group, whose MST was 16.8 months and whose 5-yr survival rate was 14.7% (Fig. 4A). Furthermore, among patients who underwent CS, OS in the CD163-low group, whose MST was not reached and whose 5-year survival rate was 63.3%, was significantly longer than that in the CD163-high group, whose MST was 24.8 months and whose 5-year survival rate was 21.5% (Fig. 4B).

## Discussion

Although chemotherapy is a standard treatment for MGC, the MST of MGC patients who underwent chemotherapy was only 13–16 months in previous studies [19]. In this study, the MST of MGC patients who underwent CS following DCS therapy reached 33.3 months, indicating that this multidisciplinary approach is a promising treatment option.

This is the first study, to our knowledge, to quantify various TIICs in pre-treatment endoscopic biopsy samples of MGC patients and investigate their predictive value for multidisciplinary treatment. Among the clinicopathological factors prior to chemotherapy, high infiltration of CD163^+^ macrophages, considered M2 macrophages, was an independent prognostic factor for OS. Additionally, pathological responders to DCS therapy showed low infiltration of CD163^+^macrophages and high infiltration of CD8^+^lymphocytes, and even in patients treated with CS, the CD163-low group showed significantly prolonged survival compared with the CD163-high group. In the present study, pathological response was an independent prognostic factor among postoperative parameters, consistent with previous reports evaluating CS for stage IV GC [20]. Pathological response is widely recognized as an important predictor of neoadjuvant chemotherapy and was the best surrogate endpoint of OS in neoadjuvant trials for GC [21]. However, the significant drawback is that this parameter cannot be clarified without surgery. One of the critical requirements for CS is a successful response to induction chemotherapy [4]. In the present study, 25 of 28 patients with CD163-low tumors were in the CS group, while 15 of 18 patients in the non-CS group had CD163-high tumors. Considering that CD163^+^ macrophage infiltrations correlates with pathologic response in the CS group, we suppose that this fact reflects that CD163-low tumors respond better to induction chemotherapy than CD163-high tumors. Those findings suggest that the prognostic value of CD 163^+^ macrophage infiltrates for multidisciplinary treatment is mainly attributable to its sensitivity to induction chemotherapy. We acknowledge that the predictive impact of CD163^+^macrophages was obtained by their measurement within pre-treatment biopsy samples, as performed in previous studies of neoadjuvant chemotherapy for esophageal and breast cancer [22, 23]. To our knowledge, this study is the first to demonstrate the efficacy of CD163^+^macrophages for the response to induction chemotherapy plus CS in MGC patients. Thus, for patients with low CD163^+^macrophage infiltration in pre-treatment biopsy samples, diagnostic imaging should be performed frequently during chemotherapy to avoid missing the optimal timing for CS, and CS should be aggressively considered as a treatment option if curative resection is deemed feasible.

TAMs, mainly composed of the M2 macrophage phenotype, have tumor-promoting activities, including supporting tumor cell proliferation, angiogenesis, and lymph-angiogenesis [19–22]. Additionally, studies have reported that a high density of TAMs infiltrating the tumor is related to a poor prognosis for several malignancies, including GC [14, 24]. Although some studies have shown that the immune system promotes the antitumor effects of chemotherapeutic agents by inducing immunogenic death of tumor cells [25], it has been suggested that chemotherapy can stimulate tumor growth by promoting macrophage-activated mechanisms, thereby inhibiting the effects of antitumor agents and inducing chemoresistance [26]. Evidence of a tumor-protective function of macrophages was shown with some antitumor drugs, platinum, 5-fluorouracil, doxorubicin, paclitaxel, and gemcitabine [27, 28]. TAMs can interpret tumor cells directly and lead to chemoresistance through the production of colony-stimulating factors (CSF)-1, increasing protease activity, inducing stemness of cancer cells through IL-6-mediated activation of STAT3 signals, and inducing enzymes involved in drug inactivation [29]. Indeed, TAMs depletion by neutralizing CSF1R improves chemotherapeutic response through decreasing the STAT3 activation in pancreatic cancer stem cells [30]. Furthermore, increasing data suggest that TAMs could also mediate chemoresistance by suppressing the CD8^+^cytotoxic T-lymphocytes that are considered key players in anticancer surveillance. For instance, DeNardo et al. reported that in breast tumors treated with paclitaxel, TAM infiltration restricted the infiltration of CD8^+^ lymphocytes and reduced their antitumor activity. Depletion of TAMs by a CSF1R antagonists combined with chemotherapy improves survival of CD8^+^lymphocytes, and consequently, the response to chemotherapy [28]. Petty et al. also demonstrated that M2 polarization of TAMs suppressed CD8^+^lymphocyte infiltration into the tumor microenvironment by inhibiting CXCL9 and CXCL10 production [11]. Thus, TAMs may contribute to chemoresistance by non-immunological and immunological mechanisms. In the present study, we demonstrated known lymphoid biomarkers of response to chemotherapy as reported in GC, including CD4^+^ and CD8^+^lymphocyte infiltration [31]. However, these lymphoid infiltrates were less significantly correlated with OS compared with CD163^+^macrophage infiltration. The density of these lymphoid infiltrates varied according to the stage of the disease, with distant metastatic disease having a relatively "colder" TME. Therefore, it is possible that less immunogenic tumors, such as earlier-stage tumors, potentially account for the different responses to chemotherapy in the patients enrolled in this study.

Another intriguing finding in our study was that high infiltration of CD163^+^macrophages correlated with higher NLR and lower LMR. Evidence indicates that the preoperative serum systemic inflammatory response, including NLR, LMR, and PLR, has also prognostic value in many cancers, including GC. Nakamura et al. reported that NLR was an independent predictor of achieving CS in stage IV GC patients [32]. Several additional studies have shown an association between TIIC as a local immune response and serum systemic inflammatory response markers [33]. Although systemic inflammatory response markers were not identified as a prognostic factor for OS in our study, their association with macrophage infiltration warrants further investigation.

As prospects for the future, M2 macrophages may be a promising therapeutic target as well as a predictor of therapeutic efficacy. Several clinical trials have reported significant synergy with chemotherapeutic agents, but the efficacy of single agents is limited [34]. Macrophage-targeted therapies are expected to improve the therapeutic efficacy of existing conventional chemotherapy via activation of anti-cancer immunity, especially for patients with high infiltration of TAMs into GC tissue.

There were several limitations in our study. First, this study was retrospective in design and included a relatively small number of patients. Second, we did not analyze the molecular mechanisms underlying the antitumor immune response induced by cytotoxic agents. Third, there were certain limitations in the analysis of immunohistochemical markers for TAMs. Regarding the sub lassification of macrophages, no surface markers have been established that clearly distinguish M1 from M2 macrophages. We examined CD163 as a representative M2 marker in this study because the mechanistic role of CD163 in macrophage M2 polarization is best understood [35].

In conclusion, the present study confirmed that pre-treatment CD163^+^macrophage infiltration was a pivotal biomarker for predicting the prognosis and pathological response to multidisciplinary treatment among a variety of immune cells in MGC. Patients with low CD163^+^macrophage infiltration are more likely to achieve curative resection and to benefit from CS. The introduction of CD163^+^ macrophage quantification in clinical practice is very useful in determining indications for CS as a personalized medicine to eliminate unnecessary invasive treatment and to maximize the multidisciplinary therapeutic effect.

## Data Availability

The datasets analyzed during the current study are not publicly available due to limitations of ethical approval involving the patient data and anonymity but are available from the corresponding author on reasonable request.

## Abbreviations

MGC: Metastatic gastric cancer
CS: Conversion surgery
DCS: Docetaxel plus cisplatin plus S-1
TIICs: Tumor-infiltrating immune cells
TILs: Tumor-infiltrating lymphocytes
TAMs: Tumor-associated macrophages
NLR: Neutrophil-to-lymphocyte ratio
LMR: Lymphocyte-to-monocyte ratio
PLR: Platelet-to-lymphocyte ratio
MST: Median survival time
OS: Overall survival
CSF: Colony-stimulating factor
